# Potentially Treatable Disorder Diagnosed *Post Mortem* by Exome Analysis in a Boy with Respiratory Distress

**DOI:** 10.3390/ijms17030306

**Published:** 2016-02-27

**Authors:** Valentina Imperatore, Maria Antonietta Mencarelli, Chiara Fallerini, Laura Bianciardi, Francesca Ariani, Simone Furini, Alessandra Renieri, Francesca Mari, Elisa Frullanti

**Affiliations:** 1 Medical Genetics, University of Siena, Policlinico Le Scotte, Viale Bracci 2, 53100 Siena, Italy; imperatore2@student.unisi.it (V.I.); mariaantonietta.mencarelli@unisi.it (M.A.M.); fallerini2@unisi.it (C.F.); bianciardi19@unisi.it (L.B.); francesca.ariani@unisi.it (F.A.); elisa.frullanti@dbm.unisi.it (E.F.); 2 Genetica Medica, Azienda Ospedaliera Universitaria Senese, 53100 Siena, Italy; 3 Department of Medical Biotechnologies, University of Siena, 53100 Siena, Italy; furini.simone@gmail.com

**Keywords:** exome sequencing, myasthenia, perinatal death, complex disorder

## Abstract

We highlight the importance of exome sequencing in solving a clinical case of a child who died at 14 months after a series of respiratory crises. He was the half-brother of a girl diagnosed at 7 years with the early-onset seizure variant of Rett syndrome due to *CDKL5* mutation. We performed a test for *CDKL5* in the boy, which came back negative. Driven by the mother’s compelling need for a diagnosis, we moved forward performing whole exome sequencing analysis. Surprisingly, two missense mutations in compound heterozygosity were identified in the *RAPSN* gene encoding a receptor-associated protein with a key role in clustering and anchoring nicotinic acetylcholine receptors at synaptic sites. This gene is responsible for a congenital form of myasthenic syndrome, a disease potentially treatable with cholinesterase inhibitors. Therefore, an earlier diagnosis in this boy would have led to a better clinical management and prognosis. Our study supports the key role of exome sequencing in achieving a definite diagnosis in severe perinatal diseases, an essential step especially when a specific therapy is available.

## 1. Introduction

Next-generation sequencing (NGS) techniques, such as exome sequencing, are now able to examine a large number of genes in a single reaction and perform high-throughput analyses of the patient’s genome, providing rapid identification of mutations involved in the onset of genetic diseases [1]. This makes these new techniques extremely important to the study of complex phenotypic disorders [2].

Congenital myasthenic syndromes (CMS) are a heterogeneous group of disorders characterized by abnormal synaptic transmission at the neuromuscular junction [3]. At present, 25 genes have been associated with CMS [4]. Here, we report the efficacy of exome sequencing approach in detecting two missense causative mutations in the *RAPSN* gene in a child with a complex clinical phenotype who died at 14 months of age because of respiratory crises. *RAPSN* encodes for the receptor-associated protein of the synapse (rapsyn). Rapsyn plays a central role in acetylcholine receptors’ (AChRs) clustering and stabilization during the formation of the neuromuscular synapse [5]. Notably, we therefore underline the essential role of whole exome sequencing application in reaching a definitive diagnosis, and we highlight the importance of this key step in order to define a proper therapeutic approach in the proband.

## 2. Results

### 2.1. Case Description

**Figure 1:**
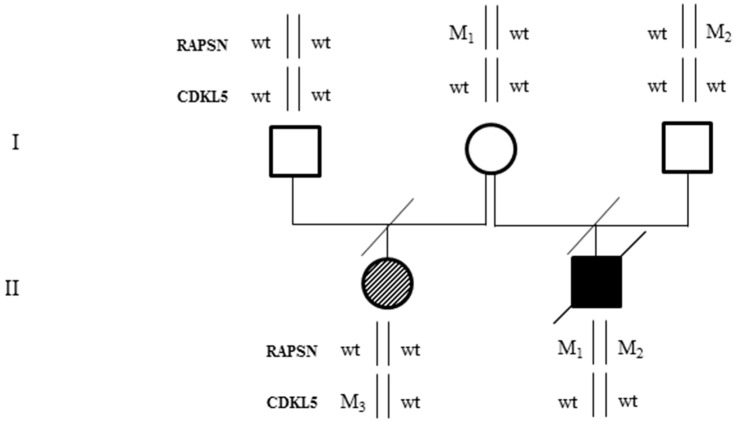
Family’s pedigree. The proband is represented by a black square. He is affected by a form of congenital myasthenia and harbors two mutations in compound heterozygosity in *RAPSN* gene: p.Asn88Lys (M_1_) and p.Val165Met (M_2_). The half-sister, represented by a striped circle, is affected by Rett syndrome with early onset seizures and harbors the p.Glu364* (M_3_) *CDKL5* pathogenic mutation.

The proband (II-2, Figure 1) was born at the end of an uneventful pregnancy by Caesarean section for fetal bradycardia. No consanguinity was reported between his parents (Figure 1).

Cytogenetic analysis performed on chorionic villus sampling revealed a normal 46,XY chromosomal constitution. At birth the weight was 4.250 g (>90° centile), the length was 53 cm (>90° centile), the occipital frontal circumference was 37.5 cm (>90° centile), and the Apgar index was 7 and 9 at the first and the fifth minute, respectively. The baby was hospitalized for three months for hypotonia with feeble cry, and sucking and swallowing incoordination, which required in some circumstances nasogastric tube feeding, and crises of apnea and cyanosis associated to laryngeal stridor, which often resolved with administration of oxygen. It was also noted that episodes of apnea occurred many times during the day and that the baby also presented breathing problems with abundant mucus production. At physical examination no peculiar features were ascertained. Lactate, pyruvate, and ammonium dosage both in the blood and in the cerebrospinal fluid were in the normal range. Brain MRI (magnetic resonance imaging), video-EEG (electroencephalogram), and brainstem auditory evoked potentials were normal. Mitochondrial DNA analysis did not reveal any alteration. A pharmacological therapy with phenobarbital was attempted even if video EEG did not document abnormalities during the crises. The proband died at 14 months of age because of prolonged apnea.

His elder half-sister (II-1, Figure 1) was born by natural delivery and started to suffer from epilepsy at 45 days of age. She presented with psychomotor development delay; she acquired the sitting position at nine months of age and started to walk independently at four years of age. A *de novo* p.Glu364* mutation in heterozygous state was detected by molecular analysis of the *CDKL5* gene only after her brother’s death. The *CDKL5* mutation was not found in the proband’s DNA after *post mortem* analysis.

### 2.2. Whole Exome Sequencing Analysis

**Figure 2:**
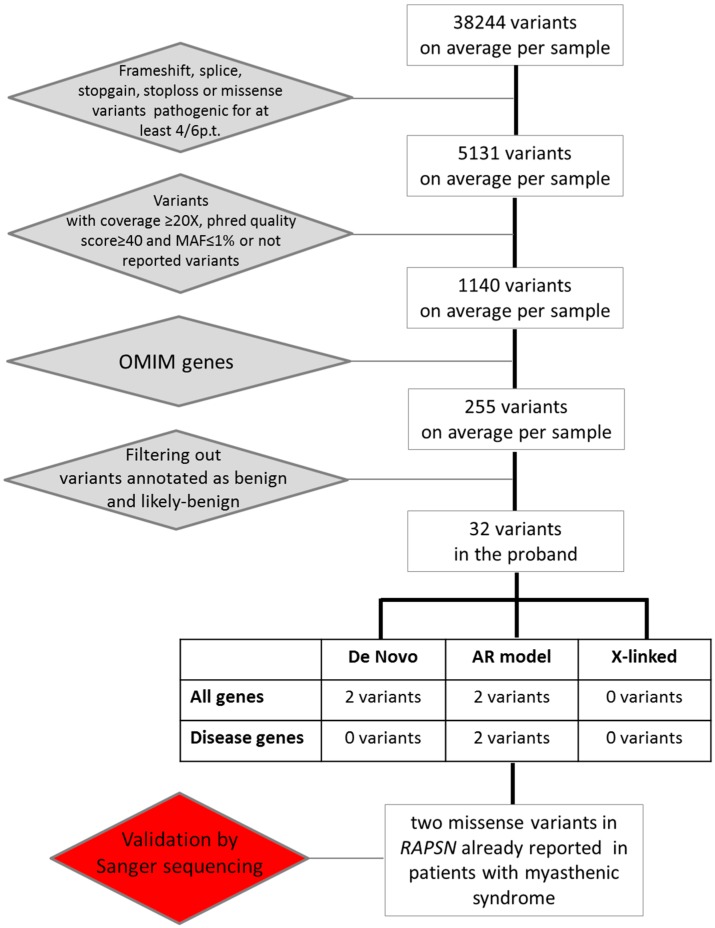
Flowchart illustrating filtering process and variants selection used to identify pathogenic variations.

Given the complexity of the clinical spectrum, we decided to perform whole exome sequencing on DNA samples from both the autoptic material and parent’s blood samples. Exome sequencing yielded a total of 38,244 genetic variants on average for each sample for further investigation (Figure 2).

We filtered for variants that were either loss of function (frameshift, splice, stopgain, or stoploss mutations) or missense variants predicted to be damaging by at least four out of six pathogenic prediction tools, and identified around 5131 variants per sample. Among them we selected the variants with coverage ≥20× and phred quality score ≥40, and with a frequency <1% or not reported. In this way, we obtained an average of 1140 variants per sample. Then we applied another filtering step by selecting the variants in OMIM genes (255 variants per sample). Out of these 255 variants, we excluded 223 variants that were annotated as benign or uncertain, resulting in 32 variants in the proband. We decided to focus our attention on variants lying in disease genes consistent with *de novo*, autosomal recessive, or X-linked mode of inheritance according to the filtering criteria described in the experimental section. We detected a total of two variants fitting the criteria for an autosomal recessive mode of inheritance. No variant consistent with either a *de novo* or an X-linked pattern of inheritance was identified (Figure 2).

**Figure 3:**
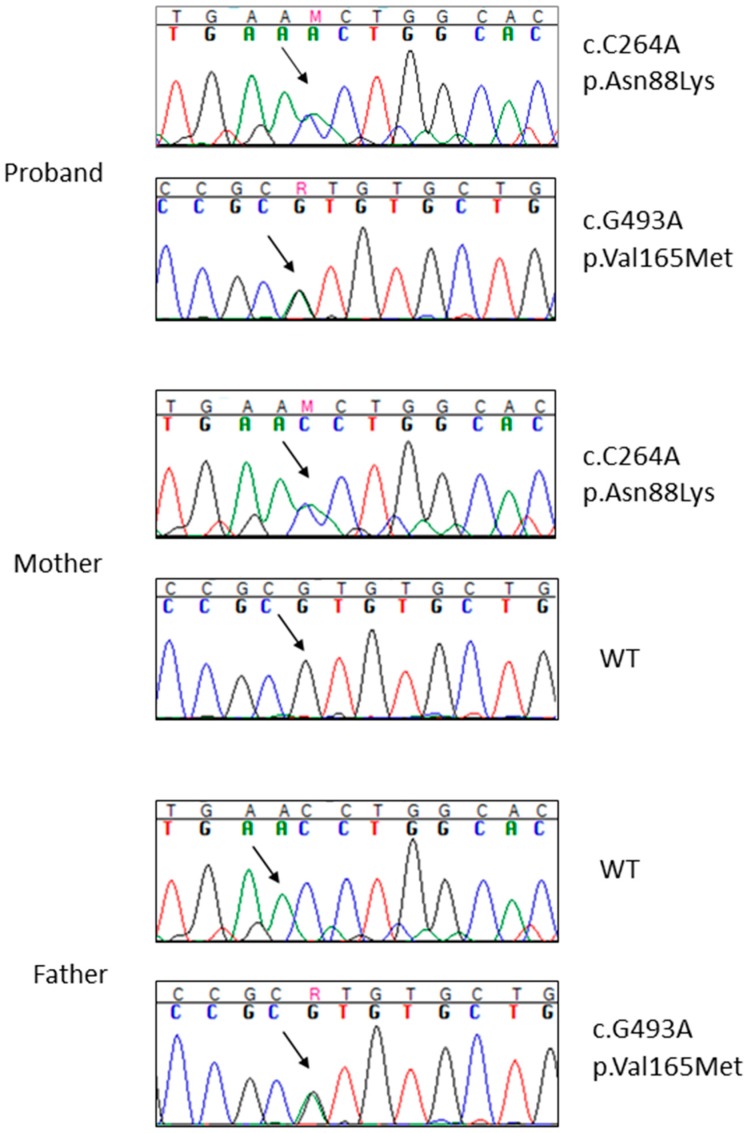
Sanger sequencing of the two *RAPSN* variants (p.Gln88Lys **above** and p.Val165Met **below**) in the proband and in his parents. The arrows indicate the mutation site. Different colors identify the four different nucleotides (A = green, C = blue, G = black, T = red). Wt = wild type.

These are two missense mutations occurring in the *RAPSN* gene (OMIM #601592) on chromosome 11: c.493G>A, p.Asn88Lys and c.264C>A, p.Val165Met (NM_005055, exon2). Both variants were present in the proband in compound heterozygosity, one inherited from the father and the other from the mother (Figure 3). The variants were validated by Sanger sequencing in the proband and his parents (Figure 3).

## 3. Discussion

In this paper, we report the use of an exome sequencing approach to unravel the genetic cause of a difficult case of congenital disease—where the phenotype overlapped with several syndromes, most of which are genetically heterogeneous—in a proband who died at 14 months of age. He was the half-brother of a girl who was diagnosed with a Rett variant due to a *de novo* *CDKL5* mutation after his death. The absence of a diagnosis in his sister when the boy was symptomatic has hampered the consideration of two distinct genetic events in the two siblings.

Driven by the mother’s compelling need for a diagnosis, we performed exome sequencing analysis that successfully identified two missense mutations (p.Asn88Lys and p.Val165Met) in the *RAPSN* (receptor-associated protein of the synapse) gene, leading to a *post mortem* diagnosis of autosomal recessive congenital myasthenic syndrome-11 (CMS11; OMIM#616326) in our proband. This condition belongs to the group of congenital myasthenic syndromes characterized by abnormal synaptic transmission at the neuromuscular junction and by a very wide clinical phenotypic expression from early onset cases with respiratory failure to adult onset milder forms [6]. CMS11 has itself a broad clinical variability ranging from neonatal severe hypotonia with poor suck and cry, episodic apnea, and respiratory difficulty, often requiring assisted ventilation, to mild muscle weakness [7]. However, this clinical presentation, especially when severe, overlaps with other genetic disorders like metabolic conditions, congenital myopathies, and muscular dystrophies.

The causative *RAPSN* gene encodes a 43-KD (Kilodalton) protein that connects AChR at the neuromuscular junction, thereby stabilizing AChR clustering [8]. Mutations in this gene are associated with AChR deficiency [7]. Several missense mutations in *RAPSN* have been reported in patients with CMS11 [9]. Among them the p.Asn88Lys is one of the most frequent pathogenic mutations [9]. Patients homozygous for the p.Asn88Lys have the mildest symptoms [10], while patients compound heterozygous for p.Asn88Lys, such as our proband, have a severe clinical presentation [9,11], suggesting that the second mutated allele may largely determine the phenotype.

The other p.Val165Met missense mutation identified in our patient has also been reported in the literature and two unrelated sporadic cases have been described bearing the same combination of mutations as in our proband [12,13]. Both patients presented a very severe and early onset disease with major pharyngeal and respiratory involvement, as in our case.

Both mutations lie in domains involved in rapsyn self-association: the p.Asn88Lys and the p.Val165Met variants are located in the TPR3 and TPR5 domains, respectively [12]. *In vitro* studies demonstrated that Lys88-rapsyn-mediated AChR clusters are less stable and reduced in number, with respect to those mediated by the wild-type protein [9].

CMS is currently treated with cholinergic agonists, namely pyridostigmine and amifampridine, adrenergic agonists such as salbutamol and ephedrine, and long-lived open-channel blockers of the acetylcholine receptor ion channel such as fluoxetine and quinidine. Given the different mechanism of action of these drugs, different treatments can have distinct effectiveness on different types of CMS, making molecular diagnosis essential for the correct choice of a proper therapy [6]. It is heartbreaking knowing that patients with CMS11 and compound heterozygous mutations in *RAPSN* gene improved motor strength and movement with anticholinesterase treatment [9,10] and that our patient would have benefited from this therapy if he had been diagnosed earlier.

Herein, whole exome sequencing has allowed us to identify a causative mutant gene and this finding has led to the clinical diagnosis of a treatable disease. However, in our case the treatment was no longer possible since the diagnosis was reached only *post mortem*. A different ending was described for a 20-month-old boy who presented a nonspecific phenotype, as in our case, and was found to have a diagnosis of CMS11 after exome sequencing analysis [11]. In this case, thanks to this new genetic diagnostic approach, the baby was finally treated with pyridostigmine, which resulted in a dramatic improvement of his clinical status [11]. Therefore, we underline the importance of performing exome sequencing in newborns with an uncertain diagnosis for whom prompt treatment is extremely important in order to definitely reach a correct diagnosis, guarantee an early treatment, and avoid perinatal death. This concept is especially crucial in those cases where the clinical signs are not uniquely attributable to a specific medical condition or group of conditions.

Our work is in line with recent evidence emerging from clinical practice that supports the use of NGS techniques, namely exome sequencing for all newborns with complex diseases [14]. Indeed, geneticists of Children’s Mercy Hospital (Kansas City, MO, USA) have been able to reach a diagnosis in more than 50% of sequenced infants with suspected genetic disorders and to recommend changes in therapeutic treatment in about half of them [14]. Thus, we strongly recommend that the use of exome sequencing become a common routine step in clinical practice rather than an exception, especially in the presence of complex diseases often misdiagnosed or under-diagnosed.

## 4. Experimental Section

### 4.1. Samples and DNA Extraction

Parents provided and signed a written informed consent at the Medical Genetics department of the University of Siena, Italy for exome sequencing analysis, clinical data usage, and the use of DNA samples from the tested individuals for both research and diagnosis purposes. Genomic DNA from the parents was isolated from EDTA peripheral blood samples using a QIAamp DNA Blood Kit according to the manufacturer’s protocol (Qiagen^®^, Hilden, Germany), while the proband’s genomic DNA was obtained from autoptic material.

### 4.2. Whole Exome Sequencing

Exome sequencing was performed on genomic DNA samples of the proband and both parents using the Life Technologies Ion Proton sequencer (Life Technologies, Carlsbad, CA, USA). Ion Torrent Sequencing technology is based on the new AmpliSeq™Exome method, which allows for improved parameters related to precision, uniformity, ease of use, and time to results compared to all other existing enrichment systems. This system enables >92% of bases covered ≥20×. Sample preparation and sequencing were performed with AmpliSeq™Exome strategy, following the manufacturer’s protocol (Life Technologies). The library preparation was performed using the Ion AmpliSeq™Exome Kit (Life Technologies), which allows us to target ~33 Mb of coding exons plus 15 Mb of flanking regions for a total of ~58 Mb, in total more than 97% of the coding regions described by Consensus Coding Sequences (CCDS) annotation, using 12 primer pools for highly specific enrichment of exons within the human genome. Taking advantage of a barcode system, three samples were loaded together in a single run and sequenced.

Data analysis was performed with Torrent Suitet™ Software v3.6.2 (Life Technologies). The provider generated at least 30× effective mean depth per sample. Using specific parameters, we were able to remove the adaptors’ contamination and low-quality sequences, so the total amount of clean data was mapped to the UCSC/hg19 reference genome. Indel and variant calls were made using GATK version 2.7 ( Broad Institute, Cambridge, MA, USA) (and its recommended parameters) [15] and then the variants were also annotated against external datasets, including 1000 genomes and dbSNP.

An additional filtering procedure was then implemented for retrieving variants with a potential detrimental impact on protein function, *i.e.*, truncating, splice site variants, and missense mutations. Pathogenicity of missense variants was assessed according to the following six predictive tools: SIFT [16], PolyPhen-2 [17], Mutation Taster [18], Mutation Assessor [19], FATHMM [20] and GERP [21] combined algorithm, and LRT [22].

A filtering step was performed in order to identify *de novo* and recessive and X-linked mutations for variants predicted to be deleterious. For the X-linked transmission pattern, a score equal to 1 was given if the variant was on chromosome X found in the proband and present in heterozygosis in the mother; for the recessive pattern of transmission, a score of 1 was given if the variant was present in homozygosis in the proband and in heterozygosis in both parents. A score of 2 was given if two different variants were found in compound heterozygosity in the proband and the parents were heterozygous for each variant.

### 4.3. Sanger Sequencing

The two RAPSN variants were validated by Sanger sequencing using ABI Prism 310 genetic analyzer (PE Applied Biosystems, Foster City, CA, USA) and data were analyzed with Sequencher software V.4.9 (Gene Codes, Ann Arbor, MI, USA). The first region (246 bp) and the second region (263 bp) were amplified using the couple primers RAPSN-F (5′-tcagagtccaggctgagc-3′) and RAPSN-R (3′-TTGCCCATGCTCAGGCTGAC-5′); and RAPSN-F (5′-TGCCTGGTACCAGGGCAG-3′) and RAPSN-R (3′-gtgtgtgcctcatgagagg-5′), respectively.

### 4.4. Mutation Nomenclature

All mutations are described according to Human Genome Variations Society (HGVS) [23,24]. Nucleotide numbers are derived from the cDNA sequence of *RAPSN* (GenBank accession no. NM_005055).
